# Learning in a time of crises: The learning experiences of 5th year medical students at the University of KwaZulu-Natal during the COVID-19 pandemic in 2020

**DOI:** 10.4102/phcfm.v13i1.3002

**Published:** 2021-09-06

**Authors:** Andrew J. Ross

**Affiliations:** 1 Discipline of Family Medicine, College of Health Sciences, University of KwaZulu-Natal, Durban, South Africa

**Keywords:** COVID-19, learning, access, online teaching, South Africa

## Abstract

**Background:**

The coronavirus disease 2019 (COVID-19) global pandemic led to the closure of the University of KwaZulu-Natal (UKZN) in March 2020 and the migration to online teaching for all students, with modules and assessments being modified to accommodate the new form of teaching.

**Aim:**

The aim of this research was to assess the 5th year students’ experience of emergency on-line learning during the Family Medicine module, which was adapted in response to the pandemic.

**Setting:**

The research was conducted among 5th year medical students at the University of KwaZulu-Natal in December 2020.

**Methods:**

A questionnaire was used to assess the experiences of the 5th year MBChB students of on-line learning.

**Results:**

Of the 256 students, 43.8% completed the questionnaire; 43 (38.4%) spent lockdown in an urban area; 30 (26.8%) in semi-urban and 39 (34.8%) in rural area; 34 (30.4%) always had internet connectivity; 41 (36.6%) mostly or often; and 28 (25%) sometimes or occassionally whilst 9 (8%) did not have any connectivity. Despite data bundles being provided by the UKZN, 47 did not have access to sufficient data for their academic needs. Only 35 students felt that their environment during lockdown was conducive to online learning and 71 students were not in a good headspace to engage with online learning.

**Conclusion:**

Whilst there are undoubted advantages of online teaching and learning in terms of access and reach, this potential is limited by structural and economic factors. These issues had a major impact on the ability of students to engage with this form of instruction.

## Background

The year 2020 was an extraordinary year, with challenges never faced before across all facets of life including teaching and learning at schools and universities across the globe. In March 2020, the World Health Organization (WHO) declared the coronavirus disease 2019 (COVID-19) to be a global pandemic, with the South African (SA) president, Mr Ramaphosa, announcing that a national lockdown would start on 27 March 2020, in an attempt to ‘flatten the curve and to reduce the impact of the pandemic on the country’s healthcare system’. Educational institutions in Africa and SA were not spared from the impact of COVID-19, and on Sunday 22 March 2020 the Vice Chancellor at the University of KwaZulu-Natal (UKZN), Professor Poku, announced that the institution would be on lockdown with immediate effect, and that all students were required to vacate the residences and leave the university.

Following this decision, all teaching had to be provided online, which in this context was considered to be remote teaching and learning, for which the Internet would be used to deliver the content. A number of courses were held on how to transition to this format, and staff were also taught how to better use the learning management platform (Moodle is used at the UKZN) for online teaching, learning and assessment.^1^ Numerous documents were circulated to the UKZN staff about the need for online remedial courses, how to create a blended approach to teaching and the need to submit a Teaching and Learning Plan during the related restrictions^2^ to ensure that students would complete the 2020 academic year.^3,4^ Modules were rewritten to accommodate online teaching and changes in assessments. In addition, the UKZN made data packages available to students and tried to ensure that they had access to suitable devices (‘leave no one behind’).^5^

The change in teaching platform also affected the medical programmes run on the UKZN’s various campuses. The CMED5PC (5th year Family Medicine module) module is a 6-week Family Medicine Integrated Primary Care (IPC2) module for the 5th year medical students. In this module, groups of ± 40 students are allocated to a district hospital where they rotate through four clinical domains, namely accident and emergency, medical outpatients, HIV clinic and general practitioner (GP) practice. Following Professor Poku’s announcement, all teaching and clinical placements were suspended, and students were required to only engage with online learning. To enable student learning during the lockdown, all lectures normally given during the module were recorded and loaded onto the Moodle learning platform, whilst additional online learning opportunities were provided in the form of access to Ambos (an international learning management system), and students were encouraged to complete portfolio tasks online.

A UKZN student access report in April 2020 that was conducted with 20 297 respondents reported that 78% had access to smartphones and 73% to mobile data,^6^ suggesting that many were equipped to access online teaching. From 8 to 12 June 2020, the IPC2 course content was covered online for all the 5th year MBCHB students. However, despite the assurances that the vast majority of the UKZN students had access to reliable Wi-Fi and suitable devices with which to log on, a maximum of 160 of the 254 students registered for IPC2 logged on each day to the zoom teaching, with only ± 80 still being engaged at the end of the day. Although there may be a number of reasons for this, informal conversations with students suggested that many did not have suitable devices or access to reliable internet connections and/or were in a home environment that was not conducive to online learning.

The MBCHB programme at the UKZN has a pre-clinical (years 1–3) and a clinical component (years 4–6), the latter requiring clinical teaching and interaction with patients. The UKZN agreed that the medical students in their clinical years could return to the university in July 2020, as per the guidelines provided by the National Department of Higher Education and Training,^7,8^ with the 5th year students returning on 20 July 2020. Although the main motivation for medical students returning to campus was to allow them to engage in clinical teaching (which could not be provided online), it also ensured that students had access to reliable Wi-Fi and an environment conducive to learning. By the time the students returned to campus, the IPC2 module had been reduced from six to 4 weeks, GP rotation had been removed, students were required to attend clinical placements on alternative days, Zoom tutorials were provided on the days when not on the clinical placement and assessments had been modified. The changes were to enable some clinical exposure, continued teaching and assessment of students at the end of the module.

However, given the speed with which the change occurred to online teaching, little is known about the learning experiences of students during the lockdown. The aim of this study was therefore to understand the learning experiences of the 5th year medical students who did the Family Medicine IPC2 module at UKZN in 2020 during the COVID-19 pandemic. It is anticipated that insights gained from this study will lead to improvements in the online teaching and learning experience of students at the UKZN.

## Methods

This was a descriptive, qualitative, cross-sectional study that used an online questionnaire. The study population was all the 5th year medical students who completed the IPC2 module in 2020, with no exclusion criteria. The students were contacted via email in December 2020 and asked to complete an online anonymous questionnaire about their learning experiences during the 2020 academic year. The questionnaire was developed by the researcher and consisted of the following sections: demographics, connectivity (the Internet, devices, data, electricity), environment during lockdown, participation in Family Medicine teaching during lockdown, completion of portfolio assignments and utilisation of teaching resources, head space during lockdown and student comments on learning during the lockdown period. The questionnaire had some open-ended questions that allowed students to comment on factors influencing their ‘head space’ and to give suggestions of how to improve online learning in general and teaching and learning in the CMED5PC module in particular.

The questionnaire was piloted amongst the 4th year medical students at the UKZN, with minor changes being made based on the feedback, which included adding a question about being in a good ‘headspace’ to engage in online learning. The 4th year students indicated that during the lockdown they were anxious, stressed, distracted and uncertain about the future, and were not always in a good headspace, which impacted on their ability to engage with the online learning material. In addition, a question on the impact of load shedding was added to the questionnaire. Data were downloaded from Google forms onto an Excel spreadsheet, cleaned and analysed descriptively by using Statistical Package for Social Sciences (SPSS) version 27 to determine central tendency, variation and associations and calculate odds ratios.

## Results

During 2020, 256 5th year students were registered for the IPC2 module, of whom 112 completed the questionnaire (43.8%). Their median age was 23 years (range: 21–31 years), 47 (42%) were male and 65 (58%) were female, 83 (74.1%) were black people, four were mixed-race people (3.6%), 23 were Indian people (21.4%) and two were white people (1.8%). For the duration of the academic lockdown (22 March 2020 – 05 July 2020), the majority of the students (107; 95.5%) went home, two stayed with friends, two were able to remain in residence and one stayed with her boyfriend. Forty-three (38.4%) students considered their place of residence during the lockdown to be urban, 30 (26.8%) semi-urban and 39 (34.8%) rural. The majority of Indian students, white students and mixed-race students were from urban areas, whilst 44.6% (*n* = 37) of the black students were from rural areas.

Regarding internet connectivity, 34 (30.4%) students always had internet connectivity, 41 (36.6%) mostly/often, 28 (25%) sometimes/occasionally and 9 (8%) students did not have any. There was a clear difference in internet access between those in urban/semi-urban and rural area, the former generally having access whereas 9/39 (23%) of the latter had no internet access (Table 1). The odds ratio of urban students having internet access was 20.5 times greater than for rural students (*p* < 0.0001). In addition, only 23% (9/39) of those living in rural areas felt that they have reliable internet access in contrast to 37/43 in urban areas (Table 1).

**TABLE 1:** Student access to Internet in urban, semi-urban and rural areas.

Internet access	Urban	Semi-urban	Rural	Total
Always	24	9	1	34
Mostly	16	13	12	41
Sometimes	3	8	17	28
No connectivity	0	0	9	9

Total	43	30	39	112

The UKZN made 25 GB of data available weekly to each student to ensure that ‘no one was left behind’ and that they would all be able to participate in the online teaching, with 104 of the 112 (93%) making use of the data bundles provided. However, 47 (42.0%) indicated that they did not have access to sufficient data for their academic needs, of whom 32 (68%) were able to purchase additional data, at an average cost of R120.00 per week. The odds ratio of urban students having access to sufficient data was 5.6 times greater (*p* = 0.0006) than for rural students.

Online teaching is dependent upon many factors, including internet access, availability of a suitable device and electricity. During their time away from the UKZN, 84 (75%) of the 112 students had access to a laptop that could connect to the Internet and 104 (92.9%) had access to a smartphone or tablet, whilst four had access to neither. During the lockdown in 2020, ESKOM (National provider of electricity in South Africa) experienced challenges with supplying electricity to South Africa with 62.5% (*n* = 70) of students having been negatively impacted by the load-shedding events. Although most of these students experienced load shedding occasionally, this was sufficient to disrupt their studies.

Studying online under challenging circumstances requires a supportive environment, with 35 (31.3%) students having felt that the environment where they were during lockdown was conducive to this form of instruction. More rural than urban students felt that their environment was not conducive to studying (Table 2), as indicated by 77 (68.8%) of the 112 students. The reasons given included (they could give more than one response): required to help around the house (55/77 – of whom 23/32 [72%] were men and 32/45 [71%] were women), no space for them to work (35/77), their house was crowded (25/77) and the house was noisy (48/77). In addition, 58 students indicated that family members were supportive but did not understand their need to study, whilst four mentioned problems with connectivity.

**TABLE 2:** Environment conducive to online learning: a comparison between urban and rural students.

Environment conducive to online learning	Urban	Semi-urban	Rural	Total
Yes	29	3	3	35
No	14	27	36	77

**Total**	**43**	**30**	**39**	**112**

During lockdown, the online learning platform (Moodle) was modified to enable students to complete a number of portfolio assignments and participate in online teaching and learning (family medicine [FM] had a week in June 2020), although only 79/112 (70.5%) of those students who completed the questionnaire participated in this activity.

Many (20/33, 60%) of the students who were unable to participate in the IPC2 online teaching were from rural areas, compared with only 5/33 (15.1%) being from urban areas (Table 3). The reasons given included having no internet/poor internet speed at the time of the training (17/33, 51.5%), no data (14/33, 42%), device issues (6/33, 18.1%), home environment not conducive to participating (19/33, 57.6%), doing another module (1/33, 3%) and overloaded with work given over a short period of time (1/33, 3%).

**TABLE 3:** Student participation in on line learning and the completion of portfolio assignments.

Students responses	Urban (*n* = 43)		Semi-urban (*n* = 30)		Rural (*n* = 39)		Total (*N* = 112)	
	*n*	%	*n*	%	*n*	%	*n*	%
**Participated in the family medicine online teaching from 8 to 12 June 2020**								
Yes	38	88.4	22	73.3	19	48.7	79	70.5
No	5	11.6	8	26.7	20	51.3	33	29.5
**Used Ambos**								
Yes	32	74.4	12	40.0	16	41	60	53.6
No	11	25.6	18	60.0	23	59	52	46.4
**Did NDOH COVID online course**								
Yes	40	93.0	23	76.6	28	71.8	91	81.3
No	3	7.0	7	23.4	11	28.2	21	18.7
**Completed the travel medicine brochure**								
Yes	43	100.0	26	86.6	29	74.3	98	87.5
No	0	0.0	4	13.4	10	25.7	14	12.5
**Completed practice management teaching slide**								
Yes	38	88.4	24	80.0	28	71.8	90	80.4
No	5	11.6	6	20.0	11	28.2	22	19.6

NDOH COVID, National Department of Health coronavirus disease.

Of the 52 students who did not use Ambos, the reasons given included (more than one answer could be given): did not know about the platform (28/52, 53.8%), not sent instructions on how to use it (6/52, 11.5%), instructions on how to use it were not clear (16/52, 30.7%) and could not connect to the platform (10/52, 19.2%). One student stated that they did not find the platform useful, one relied on information provided on Moodle, one had problems with a device to connect with, three were saving data for lectures or had data issues and one highlighted unconducive environment issue. The reasons given for not completing the other assignments were similar (connectivity and device problems, unconducive home environment, trouble accessing assignments and busy with other things). In general, rural students were much less likely to participate in the online training, use Ambos, participate in the National Department of Health (NDOH) COVID online training course and complete the other assignments than the urban or semi-urban students (Table 3).

Only 41/112 (37.0%) of the students felt that they had been in a good head space during lockdown to engage with online teaching and learning (Table 4). The 71 students who said that they were not in a good headspace provided the following reasons (more than one reason could be given): anxiety about the future (42/71, 59.1%), uncertainty about my studies (57/71, 80.1%) and stressed about finances (15/71, 21.1%). Other responses included: stress because of challenges with internet connectivity, mental health issues, emotional stress at home, loss of family members during COVID-19, feeling hopeless and overwhelmed, feeling neglected by the university and receiving inadequate communication from the university about what was happening. In general, although not statistically significant, women felt that they were in a worse headspace than men (27/47; 57% vs. 44/65; 68%; odds ratio 1.55; *p* = 0.27), as did more rural than urban students (31/39; 79.5% vs. 19/43; 44.2%; odd ratio 4.89; *p* = 0.0015). In terms of ethnicity, 68.7% (57/83) of black, 47.8% (11/23) of Indian and 75% (3/4) of mixed-race students felt that they were not in a good head space for studying and learning during lockdown (odds ratio when comparing Indian with black students was 2.39, which was not significant, with a *p* = 0.692).

**TABLE 4:** Good head space for learning – comparison between urban and rural students.

Good head space for learning	Urban	Semi-urban	Rural	Total
Yes	24	9	8	41
No	19	21	31	71

Total	43	30	39	112

The opportunity to provide suggestions about how the online teaching could be improved was included in the questionnaire, as indicated in Table 5. Practical suggestions were made about the lecture time and having more breaks, the need for educators be trained, for students to have adequate data and how being in the residence earlier would have made a difference to their having access to the Internet (Table 5).

**TABLE 5:** Suggestions from students about how online teaching could be improved.

Areas of improvement	Student suggestions
Improve quality of lectures	‘Use the free Harvard Crimson Computer Science course [*CS50*], which provides some innovative ways of using video technology to make online lectures with an almost Netflix-level production quality [*https://online-learning.harvard.edu/course/cs50-introduction-computer-science?delta=0*]’. (P99, male, Indian person, urban) ‘Have small and easy quizzes at the end of each day/week to help recap/cement the core’. (P6, male, Indian person, urban)
Duration of lectures / breaks between lectures	‘Reduce the duration of lectures as they were very long’. (P19, male, Indian person, urban) ‘Have more breaks to give students opportunity to do home chores’. (P98, male, black person, semi-urban) ‘Take 5–10 min breaks between lectures that allow students and lecturers to stand up and feel refreshed’. (P104, male, Indian person, semi-urban) ‘Have more frequent breaks, even if it is just 5 min’. (P14, female, white person, urban)
Interaction with students / feedback to students	‘Lecturers to engage with the students’. (P65, female, mixed race person, urban) ‘Counselling, advice/guidance, one-on-one feedback sessions to improve performance’. (P111, male, Indian person, urban)
Smaller groups	‘Smaller groups rather than the whole class’. (P30, female, black person, urban)
Asynchronous teaching and learning	‘Do the lectures in my own time’. (P81, female, black person, rural) ‘Upload recorded learning videos to a drive [*allowing those with connectivity issues to download and watch them when they can*].’ (P12, female, black person, rural) ‘Uploading them to Moodle didn’t help because that only allows us to stream. Streaming on Moodle vs. Zoom makes no difference, if I have trouble connecting on the one, I won’t be able to connect on the other’. (P12, female, black person, rural)
Training for lectures	‘Every educator needs to have Zoom training’. (P10, female, white person, urban)
Access to residence	‘Online learning should rather be performed at school in order to accommodate every student’. (P106, female, black person, urban) ‘Sufficient data and a proper environment for learner to engage in online learning would be helpful’. (P101, male, black person, rural) ‘It would have been better if we were back at school’s residences where there is good internet connection and there aren’t many interruptions’. (P71, male, black person, rural)
Provision for international students	‘Arrange data for international students as other universities did’. (P9, female, black person, rural) ‘Prioritise our return to residence as no support was given’. (P9, female, black person, rural)
Help with suitable devices and data	‘Provide laptops and enough data to use for connectivity’. (P29, male, black person, rural) ‘All the students must have good internet connection, a good study environment and all the gadgets necessary for studying’. (P33, female, black person, semi-urban) ‘More data allocated during the day rather than having 10 GB daytime and 20 GB nighttime’. (P61, female, black person, rural) ‘Engage with service providers to improve the quality of internet connections’. (P32, male, black person, rural)
Not having all the block lectures at the same time	‘Having all the lectures for all the blocks at once was excessive and did not help’. (P105, female, black person, urban)
Concern that some students were being left behind	‘Online teaching favours some students and others are left behind, which leads them to fail in the block’. (P40, male, black person, urban) ‘People who had data network problems were not catered for’. (P80, male, black person, urban)
Reduce the workload	‘There was too much work and too many assignments for the time we had. These are the things that drive students to stress. I know many students who considered writing supplementary examinations because they felt they wouldn’t be ready for the exams’. (P66, female, black person, rural)
More clinical exposure	‘It’s very difficult to do a degree such as medicine online. The amount of exposure to patients was inadequate’. (P47, female, mixed race person, urban)

Many students were positive and appreciative of the effort that staff had made to ensure that the online teaching materialised. They observed that their education could continue and that the staff did well under the circumstances, as the platforms were new to everyone. They appreciated the effort that was made to find innovative ways to make learning interactive and informative (Table 6).

**TABLE 6:** Feedback from students about online teaching.

Areas of feedback	Student feedback
Positive and appreciative	‘Everyone is new to this way of teaching and I think a good job was done’. (P68, female, black person, urban) ‘There was nothing wrong with online learning – it was just unfamiliar’. (P72, female, black person, urban) ‘I think the school did the best they could’. (P67, female, black person, semi-urban) ‘Some challenges were out of everyone’s control, and hence, I believe the team did their utmost best to make online learning accessible to most’. (P21, female, black person, semi-urban) ‘Thank you to the IPC2 team for consistently finding new and innovative ways to find medical teaching methods and for their empathy for students’. (P99, male, Indian person, urban) ‘Amongst other blocks, I really appreciate the lengths IPC2 went to ensure that students receive education amid these trying times. I really thank you’. (P48, male, black person, semi-urban)
Family medicine	‘Family medicine had the best online learning strategy – more interactive and informative. It also ensured that the material was readily and easily accessible. I honestly don’t think much improvement is required’. (P61, female, black person, rural)
Ambos	‘Platforms such as Ambos are amazing and I hope they don’t expire because I get to study at my own pace when I may be on my own in the field with cattle’. (P81, female, black person, rural) ‘The multiple-choice option assessments at the end of each section were excellent in testing understanding’. (P95, male, Indian person, urban)
Doing assignments during lockdown	‘I am grateful that we were able to complete some of our assignments during the lockdown (as finishing all of them during a 4-week block could have felt overwhelming for some students)’. (P107, male, Indian person, urban) ‘Starting the assignments beforehand helped me a lot’. (P104, male, Indian person, semi-urban)

IPC2, Integrated Primary Care.

However, not all comments were positive, including too little clinical exposure, poor access to some platforms and that too much was expected of them during the 4 weeks of the module when it restarted, given that they had to attend online sessions and prepare assignments (Table 7).

**TABLE 7:** Online experience feedback from students.

Students online experiences	Student comments
Unhappy experience	‘Had a terrible experience with online learning – hopefully we can get back to physical interaction and teaching’. (P108, male, black person, semi-urban)
Little clinical exposure	‘We had very few days of exposure to the clinical setting, and I think that can be improved by adding more days in the week’. (P91, male, black person, semi- urban)
No access / poor connectivity	‘I couldn’t access some study platforms such as Ambos, COVID-19 assignment’. (P113, male, black person, rural) ‘As a result of poor Wi-Fi connection and limited data, having online sessions and working through Ambos were challenging’. (P46, female, black person, semi-urban)
Assignments excessive	‘The assignments were too much for a 4-week rotation’. (P35, female, black person, rural) ‘The time frame was short and most assignments I could not do at home, so meeting deadlines was stressful’. (P46, female, black person, semi-urban)

## Discussion

The demographics in this study are broadly in keeping with the admission policy of Nelson R Mandela School of Medicine (NRMSM) at the UKZN. The institution accepts 250 students per year, with approximately a one-third being selected from the most socio-economically disadvantaged schools (Quintile 1, 2 and 3, which are non-fee-paying schools) without racial quotas. Half of all students are selected on merit, with 20% reserved for students with prior higher education. With regard to ethnicity, 69% of places are reserved for black students, 19% for Indian students, 9% for mixed-race student, 2% for white students and 1% others.^9^ The intention of the admission policy is to ensure increased accessibility to higher education for black students and to promote economic development in line with the National Development Plan (NDP).^10^ This is in keeping with the UKZN’s Vision and Mission of social transformation through tertiary education.^11^ The role of the university is central in social transformation, as of the 7.1 million unemployed persons in SA in the first quarter of 2020, only 2.3% were graduates.^12^ Education is therefore key to developing human capital and to improving young people’s prospect of getting a job.^12^

It is important to note that 75% of the UKZN students are funded by National Student Financial Aid Scheme (NSFAS),^13^ meaning that their household income is less than R350 000.00/year, with many having an income significantly less than this.^14^ The implications of the NRMSM admissions policy and the fact that large numbers of students are funded by NSFAS include limited financial resources of students and families to meet any additional expenses (as occurred with the lockdown with the need for additional data and access to suitable devices), which are essential if students are to access online teaching and benefit from tertiary education.

As in other countries, the UKZN staff were expected to transition rapidly from face-to-face teaching to emergency online learning. Whilst additional training was provided, most training focussed on the technical ‘how to’ of online training, with little thought given to the pedagogy of the online platform, the distinctive differences between face-to-face and on-line teaching^15^ and the threats and opportunities it provided. Some of these deficiencies were identified by students, who suggested the need for shorter lectures, more regular breaks, smaller groups, greater student–staff interaction and orientation on Zoom for staff and students.

Adapting to and accessing online teaching and learning was also a challenge for students, as highlighted in this study, with many 5th year medical students struggling with access to the Internet, lack of data, unavailability of suitable devices, power cuts, living in an environment that was not conducive to learning and coping with anxiety about their future. Many of these issues are beyond the capacity of the university to respond to and require government or external support and interventions. The NDP envisioned the creation of a developmental state^10^ that was committed to the development and progressive realisation of the aspirations of the South African population. The NDP recognised the importance of internet access for education, innovation and economic activity and made the provision of high-speed broadband internet universally available at competitive prices by 2030 as one of their targets.^10^ In 2010, only 17.0% of South Africans had access to the Internet,^10^ whilst by 2018, the General Household Survey reported that 64.7% of households were able to access the Internet either at home, workplace, place of study or internet café.^16^ However, the report highlighted that only 5.6% of urban and 1.2% of rural households in KZN had internet access at home,^16^ an important figure in the context of the challenges of accessing online education during the COVID-19 lockdown when everyone was confined to their homes. In this study, 29.5% (*n* = 33) of students were unable to participate in the online training and many, particularly the rural students, struggled to complete the assignments because of a lack of access to sufficient data and reliable internet access.

However, there are undoubted advantages to online teaching and learning, as it offers flexibility to students and staff, with both synchronous and asynchronous teaching (as highlighted by the students in this study) and can dramatically increase the reach of the university as students no longer need to attend in person. University of KwaZulu-Natal staff have risen to the challenge and many have embraced online teaching and the opportunities that it provides. Prior to the lockdown, Moodle was primarily used as a repository for student assignments, but with extensive training, staff learnt how to use it for quizzes, discussion forums, peer assessments and the end of module assessments. There is a need for staff to continue to acquire skills to better utilise the online teaching platform, in keeping with evidence from other studies on the effectiveness of techniques such as shorter lectures, using a flipped classroom format, clarification rather than content and interaction via polls and breakaway groups.^1,15^ However, there were numerous challenges associated with learning how to teach online (as highlighted by the students), with many lectures simply being uploaded as voice-over power point lectures or zoom lectures, which were given with little thought regarding the pedagogy of this method of instruction.

The potential of online teaching and learning in SA is limited by structural factors, such as the lack of connectivity and a conducive learning environment and unreliable electricity supply, as well as economic factors, such as a lack of suitable devices and funds to purchase data. As with other universities, the UKZN responded to the challenge of access by making 25 GB of data available to each student each month (10 GB during the day and 15 GB at night) and negotiated with the cellular service providers to make some of the UKZN URLs, including Moodle, Researchspace, short message service (SMS) and Sharepoint, zero rated to increase student access.^17^ Twenty of the gigs provided were locked to particular UKZN URLs to limit abuse. In addition, the UKZN was able to facilitate the provision of free laptops that were paid through NSFAS for the first year students and to provide a 50% subsidy to self-funded students.^18^ Other universities were able to assist more widely with devices, with the University of Fort Hare purchasing 6800 laptops for students at a cost of R40 million.^19^ This kind of expenditure was simply not possible for many universities burdened by years of student debt, with the UKZN being one of the most indebted universities in the country, with a student debt of over R1.9 billion.^18^

However, as highlighted in this study, and despite the report that most UKZN students would be able to participate in online teaching and learning,^6^ many were unable to engage with the learning material because of (1) lack of internet access and/or (2) lack of a suitable device and or (3) load shedding and/or students living in an environment that was not conducive to learning.

Online teaching only started in July, this being 11 weeks after the March announcement of lockdown, to enable the university to ensure that suitable arrangements were made so that ‘no one was left behind’. Despite the delay, many 5th year medical students could still not participate in the online teaching, with significantly more rural students being unable to access internet in comparison with those of their urban colleagues. This has the potential to further exacerbate the economic divide between rural and urban students as far as access to resources is concerned. Amongst the 5th year medical students, 79 (70.5%) participated in the online Zoom lectures and between 80.4% and 87.5% completed the on-line assignments, despite the UKZN providing data for students to use for academic activities. Only 53.0% of the students used the Ambos online learning platform, which complemented the resources available on Moodle. Being able to use Moodle and Ambos requires internet access and a suitable device, which was a challenge, as 42.0% of the students (47/112) reported that they did not have sufficient data for their academic needs, and only 32/47 (68.0%) were able to purchase additional data, thus effectively excluding these students from participating in the online teaching and learning.

It was of concern that over 60% (71, 63.4%) reported being under considerable mental stress during lockdown. Anxiety and stress have been reported in a number of studies because of social isolation, uncertainty about the future, fear of COVID-19 infection, frustration, financial uncertainty when family members lose their jobs, lack of social support and exposure to excessive social media.^20,21^ Natural and environmental social disasters are almost always accompanied by increases in depression, post-traumatic stress disorder and mental and behavioural disorders,^22^ making it understandable why students reported not being in a good head space to engage with online learning.

In the context of COVID-19, there have been substantial increases in anxiety and depression, substance use and loneliness in the general population.^22^ A study in China amongst 4827 participants in 2020 (mean age 32 ± 10 years, range 18–85) reported a prevalence of depression of 48.3%. This was greater in those aged between 21 and 30 years than those younger than 20 years, with the prevalence of anxiety being 22.6% and the combination of depression and anxiety being 19.4%.^23^ Both figures are much higher than the national prevalence in China (depression 6.9%, anxiety 7.6%) and are consistent with other findings, where natural disasters cause an increase in mental health problems.^22,23^ A systematic review of anxiety in 17 studies amongst a population of 63 439 showed a prevalence of 31.9% (confidence interval [CI]: 27.5% – 36.7%).^24^ Although not statistically significant, amongst the 5th year medical students at the UKZN, women were slightly more likely and rural students were almost five times more likely (*p* = 0.0015) not to be in a good head space for online studying than their urban counterparts.

Students had multiple suggestions about ways to improve online learning that ranged from additional access to data and measures to train staff. However, the most common suggestion was that students be allowed to return to campus, as this would enable them to access Wi-Fi and suitable devices with which to connect, and be in a supportive learning environment. Medical students were able to return to the UKZN campus in July 2020, which resulted in all students being able to access the Internet and suitable devices.

Without a doubt, online university learning will continue, with creative solutions to the issues identified needing to be found, particularly for rural students, to enable them to engage productively with this method of instruction. Solutions, in conjunction with government, could include the development of learning hubs at schools or libraries in the community with the necessary Wi-Fi connectivity and backup generators. In addition, online apps need be developed, which would allow students to work off line and to download resource material and submit assignments when connected to Wi-Fi. As medical students need practical and theoretical training, the university needs to invest in decentralised training and longitudinal training at hospitals and clinics in rural areas. Win–win solutions such as access to university libraries, fee reductions and consultant visits need to be agreed upon so that staff at these healthcare facilities feel valued and appreciated for the role that they can play in teaching of medical students. Universities also need to ensure that staff have the necessary pedagogy to facilitate student engagement in on-line learning and that staff and students are trained and orientated in the effective use of zoom and other similar tools. This might mean shorter lectures, regular breaks, the use of breakout groups to enable greater interaction and introducing small and easy quizzes at the end of each day/week to help recap/cement the core content. In addition to the provision of data bundles, universities need to explore the possibility of accessing libraries or local schools that have internet access that the students can use. Although medical students in this study were able to return to the residences in July 2020, in the context of COVID-19, there is concern about the potential of university residences to become super COVID-19-spreading venues if all university students are allowed to return. With the change in regulations to lockdown level 1 in March 2021, the UKZN management agreed that all students could return to their residences, which allows them to access university resources.^18^ Strict COVID-19 prevention protocols,^8^ including sanitising hands and surfaces, making the wearing of masks compulsory, encouraging social distancing and the use of a mobile screening app, which students needed to complete daily prior to coming to campus, have been developed to reduce the risk of its spread on campus. As an additional measure, the UKZN must strongly motivate for vaccination for all students in an effort to reduce the risk of its spread in university residences. In addition, as students return to campus, the UKZN needs to develop a mechanism for surveillance, reporting and interventions for mental health issues, particularly for rural origin students and to ensure that the support systems are strengthened.^22^

## Strengths, limitations and opportunity for further study

A strength of this study is that 43% of the class participated in the study, giving good insights into their experiences of online learning during the COVID-19 pandemic. Despite this being a study with a single class at a single site, it has provided a number of important insights that health professional educators (HPEs) need to reflect on:

- How HPEs can develop a pedagogy of online learning, improve their understanding of what online teaching and learning can contribute and the role that it can and cannot play in the training of healthcare professionals.
- The high prevalence of anxiety amongst students should be of concern to the university and to HPEs. Proactive programmes that can identify students at risk of mental health challenges and which can provide appropriate support when and where it is needed need to be developed. In the context of isolated online learning, a new approach to identifying their stressors and mitigating them needs to be explored, given that regular face-to-face contact may not be possible.

There is a need for ongoing research on the pedagogy of online teaching and learning methods that encourage active student participation and engagement on this platform.

## Conclusion

The COVID-19 crises have highlighted the challenges faced by the 5th year medical students in engaging with online learning. This study has highlighted the need for HPEs to review how teaching and learning occur, what resources are required for students to engage in such activities and to consider how it might be performed better in the future, as additional waves of the pandemic are likely to occur in the country until herd immunity is achieved.
